# A rapid method to quantify vein density in C_4_ plants using starch staining

**DOI:** 10.1111/pce.14656

**Published:** 2023-06-23

**Authors:** Conor J. C. Simpson, Pallavi Singh, Deedi E. O. Sogbohossou, M. Eric Schranz, Julian M. Hibberd

**Affiliations:** ^1^ Department of Plant Sciences University of Cambridge Cambridge UK; ^2^ Biosystematics Group Wageningen University Wageningen The Netherlands

**Keywords:** bundle sheath, C_4_ photosynthesis, high‐throughput phenotyping

## Abstract

C_4_ photosynthesis has evolved multiple times in the angiosperms and typically involves alterations to the biochemistry, cell biology and development of leaves. One common modification found in C_4_ plants compared with the ancestral C_3_ state is an increase in vein density such that the leaf contains a larger proportion of bundle sheath cells. Recent findings indicate that there may be significant intraspecific variation in traits such as vein density in C_4_ plants but to use such natural variation for trait‐mapping, rapid phenotyping would be required. Here we report a high‐throughput method to quantify vein density that leverages the bundle sheath‐specific accumulation of starch found in C_4_ species. Starch staining allowed high‐contrast images to be acquired permitting image analysis with MATLAB‐ and Python‐based programmes. The method works for dicotyledons and monocotolydons. We applied this method to *Gynandropsis gynandra* where significant variation in vein density was detected between natural accessions, and *Zea mays* where no variation was apparent in the genotypically diverse lines assessed. We anticipate this approach will be useful to map genes controlling vein density in C_4_ species demonstrating natural variation for this trait.

## Summary statement

Preferential accumulation of starch in bundle sheath cells of C_4_ plants allows high‐throughput phenotyping of vein density.

## 1 INTRODUCTION

Photosynthesis is the basis of life on Earth and central to this process is the enzyme Ribulose 1•5‐Bisphosphate Carboxylase/Oxygenase (RuBisCO) that operates in the Calvin–Benson–Bassham cycle to fix CO_2_. However, oxygenase activity RuBisCO results in the energy‐intensive photorespiratory pathway (Driever & Kromdijk, 2013) and in some environments costs of photorespiration are thought to have driven the evolution of carbon concentrating mechanisms such as C_4_ photosynthesis and Crassulacean acid metabolism (CAM). Thus, C_3_ photosynthesis is considered the ancestral state and despite its complexity, derived states involving carbon concentrating mechanisms are thought to have evolved repeatedly (Edwards, 2019). In the case of C_4_ photosynthesis, current estimates are that it has evolved independently in more than 66 lineages (Sage et al., 2011). The vast majority of C_4_ plants evolved specialized anatomy to compartmentalise photosynthesis between two cell types. Typically, this involves the mesophyll being the site of initial HCO_3_^−^ assimilation by phosphoenolpyruvate carboxylase (PEPC) and the bundle sheath being repurposed for the Calvin–Benson–Bassham cycle. Compared with the C_3_ state, the partitioning of photosynthesis between these two cell types is associated with changes to transcriptional, posttranscriptional and also posttranslational regulatory mechanisms (Hibberd & Covshoff, 2010; Reeves et al., 2017). Different C_4_ ‘subtypes’ have been defined by the predominant use of three C_4_ acid decarboxylases in the bundle sheath (Hatch et al., 1975). Although there is growing support for the notion that species can modify the extent to which each C_4_ acid is engaged (Omoto et al., 2012; Sales et al., 2018; Sharwood et al., 2014). For some genes encoding components of the C_4_ cycle a detailed understanding has emerged such that changes in *cis* (Gowik et al., 2004, 2017; Nomura et al., 2000; Nomura, Higuchi, Ishida, et al., 2005; Nomura, Higuchi, Katayama, et al., 2005; Williams et al., 2016) or *trans* (Brown et al., 2011; Reyna‐Llorens et al., 2018) are considered the driving forces behind alterations to gene expression. In contrast, we have a more limited understanding of the genes underpinning the evolution of Kranz anatomy and how their expression has changed during the C_3_–C_4_ transition (Sedelnikova et al., 2018).

Reports of significant natural variation in traits including components of Kranz anatomy (Kolbe & Cousins, 2018; Lundgren et al., 2016; Reeves et al., 2018; Yabiku & Ueno, 2017) provide an opportunity to probe the basis of this trait using quantitative genetics. For example, if it were possible to screen vein density or bundle sheath size rapidly in populations made up of genetically diverse individuals such as Multi‐Advanced Generation Inter‐Crossing (MAGIC) populations or natural accessions designed for Genome Wide Association Study (GWAS) it should be possible to start to link genes with Kranz anatomy. This approach would be facilitated by rapid phenotyping of these traits. Significant advances have been made in phenotyping at scale for protein activity assays (Gibon et al., 2004; Sulpice et al., 2010) or the use of robotics for data collection in the field (Virlet et al., 2017). Notably, using a phenotyping facility Guo et al. (2018) rapidly analysed 51 image‐based traits in rice under drought stress. Correlating these traits with traditional drought resistance traits enabled novel genes linked to drought resistance to be identified via a GWAS. Such approaches may need support from noninvasive imaging techniques (Yang et al., 2020) and consequently, high‐throughput image processing is key to alleviate the phenotyping bottleneck associated with laborious, error‐prone manual image analysis (Moen et al., 2019). Image analysis using Convoluted Neural Networks (CNNs) has been used to map Quantitative Trait Loci (QTL) associated with stomata size and density in maize (*Zea mays*; Xie et al., 2021). Moreover, image segmentation tools have been developed to quantify vein density (Bühler et al., 2015; Dhondt et al., 2012; Parsons‐Wingerter et al., 2014), but such automated approaches have not yet replaced manual tracing (Perez‐Harguindeguy et al., 2016). Moreover, analysis of tissues such as cotyledons of *Arabidopsis thaliana* (Dhondt et al., 2012; Parsons‐Wingerter et al., 2014) can require extensive manual supervision and may not be suited for tissues with higher vein density. Although, the powerful programme LeafVeinCNN (Xu et al., 2021) enables high‐throughput analysis of traits including vein order, areole number, vein width and vein density it currently requires manual real‐time supervision of individual images and can be computationally slow due to the number of networks in use and the number of measurements being attained per image (Xu et al., 2021). Further, to our knowledge, only one tool has been developed to assess vein structure in monocotyledons (Robil et al., 2021). As C_4_ photosynthesis is found in both monocotyledons and dicotyledons it would be useful to have a tool that permits analysis of vein density in both clades.

**Figure 1:**
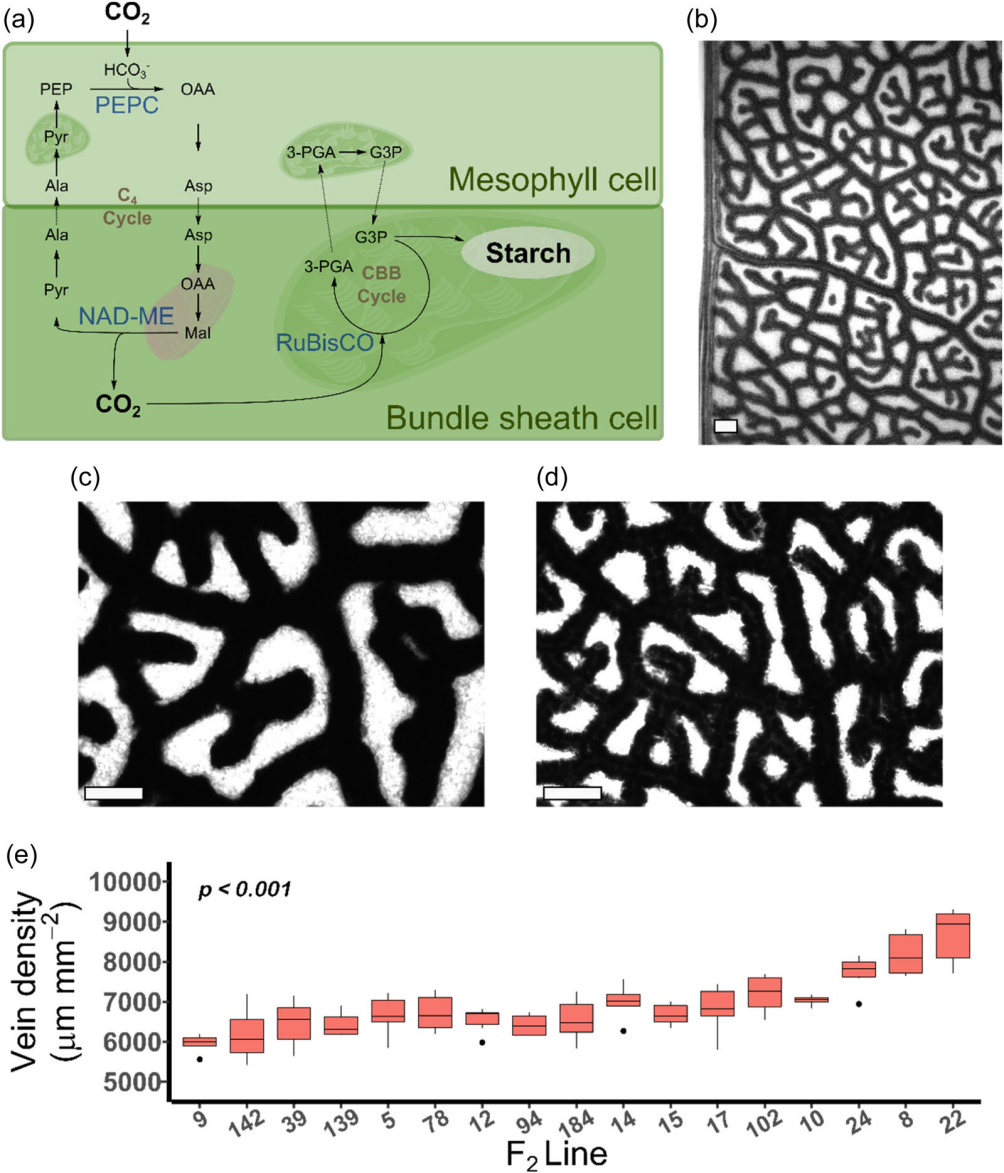
Starch staining in the bundle sheath of C_4_ *Gynandropsis gynandra* demonstrates natural variation in a diversity panel. (a) Schematic of the C_4_ pathway. Arrows indicate carbon flux. Enzymes involved in carboxylation and decarboxylation are shown in blue. Intracellular compartments represented schematically with mitochondria in pink and chloroplasts dark green. (b, c, d) Representative images of *Gynandropsis gynandra* leaves stained for starch at low magnification (b), higher magnification with low vein density (c) and higher magnification with high vein density (d). (e) Natural variation in vein density across 17 lines. Scale bars = 200 μm. Six images per plant were assessed and *p* value calculated by ANOVA. Ala, alanine; Asp, aspartate; CBB, Calvin–Benson–Bassham cycle; G3P, Glyceraldehyde 3‐phosphate; Mal, malate; NAD‐ME, NAD‐Malic Enzyme; OAA, oxaloacetate; PEP, phosphoenolpyruvate; PEPC, phosphoenolpyruvate carboxylase; Pyr, pyruvate; RuBisCO, Ribulose 1•5‐Bisphosphate Carboxylase/Oxygenase; 3‐PGA, 3‐Phosphoglycerate. [Color figure can be viewed at wileyonlinelibrary.com]

Here we aimed to test whether preferential accumulation of starch in the bundle sheath of C_4_ plants (Lunn & Furbank, 1997; Figure 1a) could be used to generate high‐contrast images so that a segmentation‐based method could rapidly assess vein density in these plants. We show that such an approach can be used to measure vein density in both monocotyledons and dicotyledons. Moreover, this allowed us to develop a computational pipeline enabling high‐contrast black‐and‐white images to be processed automatically using the image processing toolbox in MATLAB. The automated pipeline was also implemented in the open‐source Python tool. Not only does the method allow rapid phenotyping but it also showed that whilst in genotypically diverse founders of a maize population (Dell'Acqua et al., 2015) there was little variation in vein density, in the C_4_ dicotyledenous model *Gynandropsis gynandra* there is significant natural variation in vein density.

## 2 METHODS

### 2.1 Plant growth and leaf sampling

*G. gynandra* was grown under irrigated glasshouse conditions from April to July 2019. Temperatures were maintained at 24°C in the day and 20°C during the night, with artificial lighting maintaining a minimum light intensity of 300 µmol m^−1^ s^−1^ $\mathrm{µmol}\;m^{-2}s^{-1}$ and a photoperiod of 16 h light and 8 h dark. Relative humidity was 60%. Maize was grown under field conditions from April to August 2022 at NIAB. Plants were treated with pre‐emptive herbicide (Stomp® Aqua) 1 day after sowing, fertilizer (ammonium nitrate) 5 days after sowing, molluscicide (Sluxx) 1 month after sowing, and herbicide (Leystar®) 2 months after sowing. During the growing season, average temperature was 19.8°C during the day and 15.2°C during the night. Maximum and minimum daytime temperature was 39.9°C and 8.9°C and that during the night was 34.2°C and 2.9°C. Total precipitation was 89 mm and average relative humidity was 65%.

*G. gynandra* leaves were harvested over a 3‐day period from 4‐week‐old plants. They were harvested at least 7 h into the photoperiod. Briefly, the youngest fully expanded leaf was selected and tissue was harvested from the central leaflet of each leaf and immediately placed into an embedding cassette submerged along with other cassettes in 3:1 100% (v/v) ethanol:acetic acid fixative solution for 4 h. The solution was then placed in 70% (v/v) ethanol for an hour at 37°C, and then left in fresh ethanol overnight. Samples were then cleared using 5% (w/v) NaOH for 3 h at 37°C before being washed and replaced in 70% (v/v) ethanol until imaging. Samples can also be stored in 70% (v/v) ethanol before clearing with NaOH. Maize leaves were harvested over a 2‐week period as part of a larger field trial. All disks were taken from the most recently fully expanded leaf. Leaves were harvested in the field and kept in water until leaf disks were taken. One disk was taken per leaf and immediately submerged in 3:1 100% (v/v) ethanol:acetic acid fixative solution for 4 h before being transferred to 70% (v/v) ethanol solution and left overnight at 37°C. Samples were then cleared using 5% (w/v) NaOH for 6 h at 37°C before being washed with and replaced in 70% (v/v) ethanol until preparation for imaging.

*Setaria italica, Setaria viridis, Sorghum bicolor* and *Pennisetum glaucum* were sown directly onto the soil surface, covered with propagator lids to control moisture at 28°C in the absence of light for 1 day. After this, pots were transferred to a growth chamber with 12 h light, 12 h dark, maintaining a costant temperature of 28°C day and 20°C night, with ambient CO_2_ concentration and a light intensity of 500 µmol m^−1^ s^−1^ $\mathrm{µmol}\;m^{-2}s^{-1}$.

Before imaging, leaf disks were placed in Lugol's solution. Specifically, using forceps each disk was transferred to Lugol's solution and then removed immediately after submersion and placed into 70% (v/v) ethanol to minimize stain accumulation. If the stain did not accumulate rapidly the sample was replaced in Lugol's solution until it stain had been taken up. Excess Lugol's solution was washed off in 70% (v/v) ethanol. Each leaf disk was then mounted on a slide using water and imaged at a magnification of ×100 on an Olympus BX41 light microscope with a mounted Micropublisher 3.3 RTV camera (Q Imaging). Images were captured with Q‐Capture Pro 7 software.

### 2.2 Manual and automated analysis of vein density and statistical analysis

Veins were measured directly in ImageJ (Rasband, 2014) using the paint brush tool to quantify total length of veins in µm. Lengths were converted to density (µm mm^−2^) by dividing by the image's area in mm^−2^. Table 1 summarises the important MATLAB functions and their function. In addition to this, pattern recognition was used to identify commissural veins in maize (Supporting Information: Figure S3). From a subset of 102 images, 27 were also analysed using LeafVeinCNN (Xu et al., 2021). These 27 images comprised three images per plant from nine plants. LeafVeinCNN was run using default parameters consisting of three ensemble CNNs. Appropriate threshold values were determined for each image to attain optimal vein traces through segmentation. To achieve results comparable with Starch4Kranz the output value ‘VTotL’ meaning total vein length in mm, was converted to µm mm^−2^. To generate the Python version of Starch4Kranz, functions from the OpenCV (Cv2; Bradski, 2000), NumPy (Harris et al., 2020), Scikit‐image (skimage; Van Der Walt et al., 2014), SciPy (Jones et al., 2001), Matplotlib (Hunter, 2007), and PlantCV (Gehan et al., 2017) libraries were used. Important functions used are listed in Table 2.

**Table 1:** Main MATLAB base and Image Processing Toolbox functions used in Starch4Kranz.

Function	Use
rgb2gray	Converts Red Blue Green (RGB) image to greyscale
adapthisteq	Improves contrast of image using contrast‐limited adaptive histogram equalization (Zuiderveld, 1994)
blur	Blurs the image by converting each pixel to the mean of pixel value in a submatrix of 2*w* + 1 (https://uk.mathworks.com/matlabcentral/answers/472573-write-a-function-called-blur-that-blurs-the-input-image?s_tid=mwa_osa_a)
imbinarize	Replaces all pixels above a threshold to a value of 1 and makes all others 0. Threshold is based on Otsu's method (Otsu, 1979)
imcomplement	Complements an image
bwskel	Reduces binary objects to a 1‐pixel wide curved line while conserving image structure
bwmorph	Applies a particular morphological operation to a binary image. We used the ‘branchpoints’ and ‘endpoints’ operations to create two separate masks that showed the skeleton's branch points and end points, these could be used in conjunction with the find function to pinpoint their positions and lengths
bwdistgeodesic	Calculates the distance (in pixels) between defined points in a binary image. Used here to calculate the lengths of branches, that is, length between a branch point and an end point. This value was important for the trimming step of the pipeline.

**Table 2:** Main Python functions used in Starch4Kranz; citations are in the text.

Function	Use
cv2.cvtColor	Converts Red Blue Green (RGB) image to greyscale
cv2.createCLAHE	Improves contrast of image using contrast‐limited adaptive histogram equalization (Zuiderveld, 1994)
cv2.blur	Blurs image
cv2.threshold	Binarises image
cv2.bitwise_not	Complements an image
skimage.morphology.skeletonise	Reduces binary objects to a 1‐pixel wide curved line while conserving image structure
plantcv.morphology.find_branch_pts	Identifies branch points of skeleton
plantcv.morphology.find_tips	Identifies end points of skeleton

All statistical analysis were carried out in R Studio (V: 4.1.3). Figures were generated using the ggpubr (Kassambara, 2020) package. To compare manual and automated quantification methods in *G. gynandra* a subset of 17 plants from a total of 199 were selected randomly. Six images were assessed per plant such that the data set was made up of 102 images. To attain a vein density measurement for each of the 17 lines, the mean of these six images was determined. We noted that using three images yielded no significant differences in plant vein densities when comparing manual (*t* test, *p* = 0.90) and automated (*t* test, *p* = 0.85) methods. Therefore for maize, six replicates were used for A632, F7, and W153R; five for B73, CML91, H99, HP301, and Mo17; and three for B96. The total data set of 138 images was assessed both manually and automatically. For both treatments, all lines were normally distributed (Shapiro–Wilk's test, *p* > 0.05) and Bartlett's test revealed homogeneity of variance (*p* > 0.05) meaning parametric analysis was carried by analysis of variance (ANOVA). Correlation analysis was carried out using Pearson's correlation coefficient. In total, a *G. gynandra* population of 199 plants, each with five or six image replicates meaning a total of 1102 images were assessed for variation.

### 2.3 Raw script availability and code implementation

The Starch4Kranz pipeline was designed to be simple to use and can run in the background while other tasks take place. While it requires a manual check, this can be done rapidly (~5–10 s per image) as the user checks each image within a whole directory once the script has stopped running. Only three scripts are needed to run the full pipeline in MATLAB, Starch4Kranz.m contains all executable code used in the production of our pipeline, the function, blur.m contains the blurring function, and running_Starch4Kranz.m contains a short script that runs Starch4Kranz.m while saving results to a table easily transferable to software for further data analysis. For Python, just two scripts are required, Starch4Kranz.py and running_Starch4Kranz.py. Scripts ara available at https://github.com/plycs5/Starch4Kranz. To run the script in MATLAB the user must have the ImageProcessingToolbox activated and in Python they must have the dependent libraries installed into their environment. There are 14 inputs the user can supply (Supporting Information: Table S1), five of which are necessary; filename, trim_factor (see Results), pixel_length_um (the image scale), y_pixels, and x_pixels (image dimensions), while the other nine can be altered to optimise performance.

## 3 RESULTS AND DISCUSSION

### 3.1 Natural variation for vein density in C_4_ *G. gynandra* and an automated image processing tool

Staining leaves of *G. gynandra* that predominantly uses NAD‐dependent Malic Enzyme as the C_4_ acid decarboxylase clearly indicated preferential accumulation of starch in the bundle sheath (Figure 1b). We, therefore, subjected an F_2_ population of *G. gynandra* comprising 199 lines derived from a cross between parents differing in vein density to starch staining (Reeves et al., 2018). Initially, a random subset of 17 plants were stained and this indicated significant natural variation in vein density amongst the F_2_ plants (*p* < 0.001; Figure 1c–e). We reasoned that this variance in vein density between accessions provided a resource to develop and ground‐truth a semiautomated procedure to quantify vein density.

**Figure 2:**
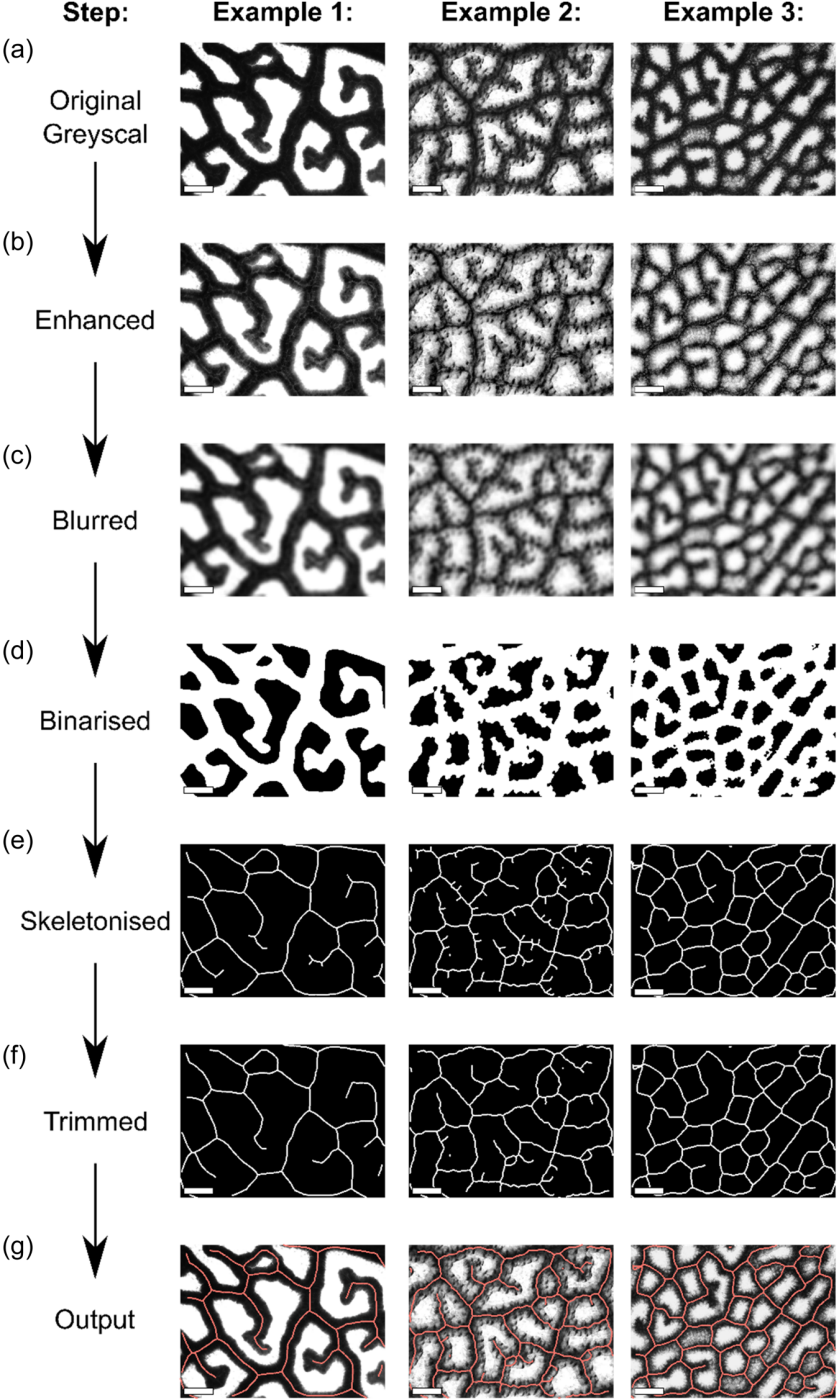
Summary of critical automation steps. (a) A greyscale image is used as input and subjected to contrast enhancement (b) followed by blurring (c), binarization (d), skeletonization (e), and trimming (f), before producing the output for the user to check (g). Scale bars = 200 µm. Skeletons have been thickened to ensure visibility. [Color figure can be viewed at wileyonlinelibrary.com]

Using MATLAB's Image Processing Toolbox (2019a) an automated pipeline, hereafter Starch4Kranz, was developed to reliably quantify bundle sheath density. This pipeline involved converting each image to greyscale (Figure 2a), enhancing the contrast to improve differences between bundle sheath strands and the background (Figure 2b) and then blurring such that a given pixel is converted to the mean pixel intensity in a submatrix of 2*w* + 1, where *w* is 20. Thus, for each pixel, its value is converted to the average of the neighbouring submatrix of 20 × 20 pixels. This smoothing resulted in pixels in each bundle sheath strand having similar values, and the same was true for background pixels (Figure 2c). Images are then binarised (Figure 2d) such that stained regions appear white (with a pixel value of 1) and the background is black (with a pixel value of 0). Skeletonisation converts all pixels with a value of 1 to curved lines a single pixel in width (Figure 2e). Lastly, these skeletonised images are trimmed such that superfluous pixels are removed (Figure 2f, Supporting Information: Figure S1). The number of pixels trimmed is defined by the user as the trim_factor, but if the programme detects a significantly larger number of superfluous pixels compared with expected vein density then the trim_factor can be calculated automatically based on the mode length of connected superfluous pixels. At this stage, bundle sheath strands are represented by a line that is one pixel thick and so the total number of pixels provides an estimate of vein length. Vein density can then be determined from the total number of pixels and the image size.

Starch4Kranz processes all images in a given directory and saves results into a table containing image name and vein density. After each image is processed a window appears containing the vein trace superimposed on the original image in greyscale (Figure 2g). Compared with approximately 11 h required to manually trace around 102 images (Figure 1e) implementing the Starch4Kranz pipeline for the same samples took 15 min.

We further implemented a Python version of Starch4Kranz so it is available as open source. The blurring method implemented by the Python version is based on openCV's blur function (Bradski, 2000) in which the kernal size was defined by the given blur_factor. To display the image, the libraries Matplotlib (Hunter, 2007), and PlantCV (Gehan et al., 2017) are required. Due to working with a large population, we adopted a sampling method that enabled rapid acquisition and concurrent fixing and clearing of large numbers of samples that could be acquired simply (see Section 2). However, due to the nature of the programme any method generating high‐contrast images of leaf veins should be compatible with Starch4Kranz.

### 3.2 Automatic quantification of high‐contrast images for vein density

**Figure 3:**
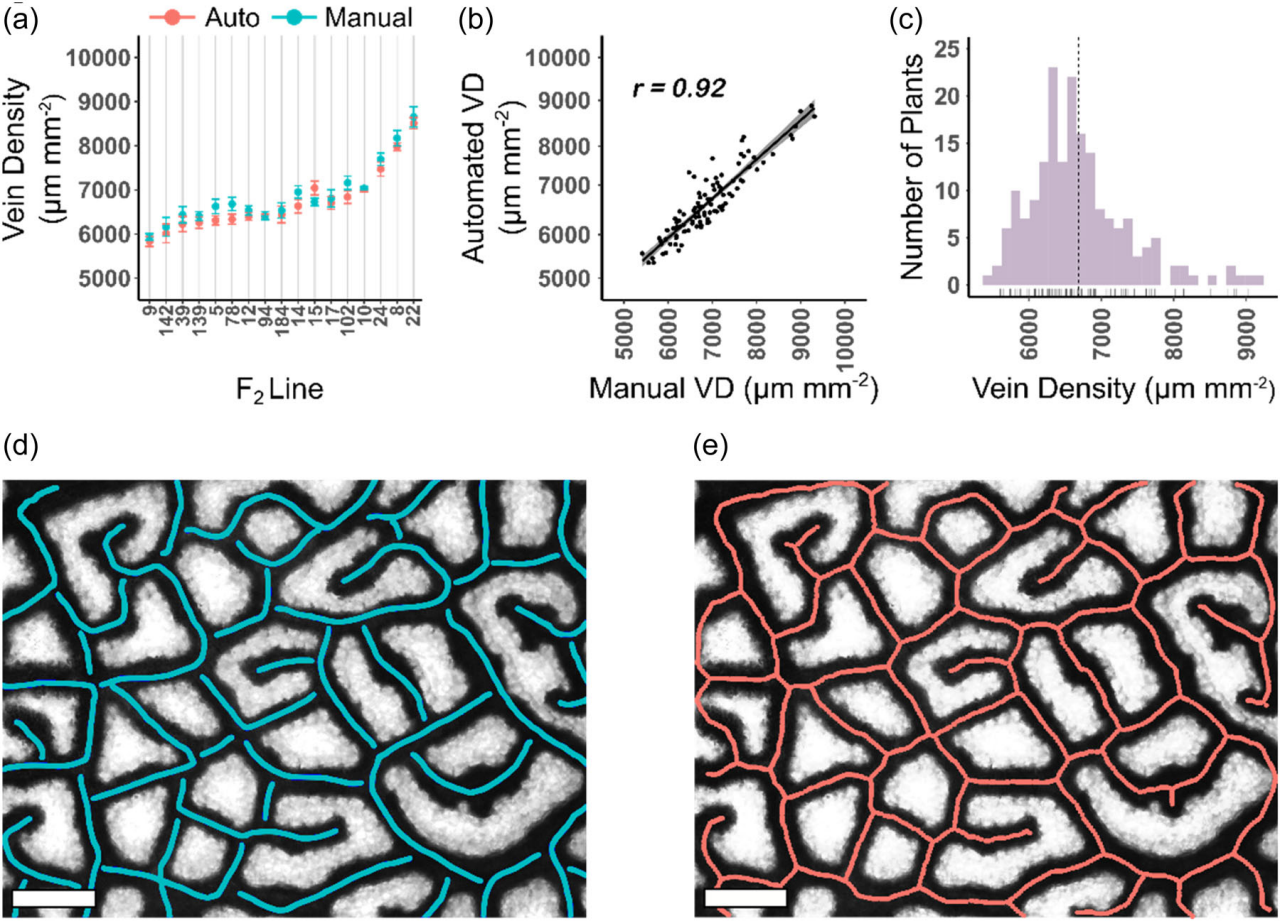
Ground truthing of automated analysis. (a) Seventeen randomly selected lines from the population comprising 199 lines were assessed both automatically (red) and manually (blue) for vein density. (b) Correlation analysis between automatic and manual quantification of vein density. Line of best fit shown with grey shading representing 95% confidence intervals. (c) Distribution of vein density across 199 plants determined using Starch4Kranz. (d) Example of a manually traced image. (e) Example of the automated output. Scale bars = 200 µm. Skeletons have been thickened to ensure visibility. *r*, Pearson's correlation coefficient; VD, vein density. [Color figure can be viewed at wileyonlinelibrary.com]

The Starch4Kranz pipeline was next implemented on data from the 17 F_2_ plants for which vein density had been determined manually (Figure 3). From 102 images, the same pattern of variation in vein density was observed (Figure 3a) and estimates from manual and automated pipelines were highly correlated (*r* = 0.92; Figure 3b). This was also the case for the Python version, which correlated strongly with manual assessments (*r* = 0.91; Supporting Information: Figure S1A), and with the MATLAB version (*r* = 0.97; Supporting Information: Figure S1B). When Starch4Kranz was applied to 1101 images, 98.5% passed the manual check. Of the 17 images that did not, manual inspection indicated they were poorly stained or the images were of poor quality. To reduce chances of poor staining, sampling late in the photoperiod is helpful—we achieved maximal staining 9 h into a 16 h photoperiod (Supporting Information: Figure S3). We compared Starch4Kranz with existing software. With PhenoVein (Bühler et al., 2015) installation requirements meant the software was awkward to implement, but also that it needed extensive manual input into each image. The requirement for manual inputs meant direct comparison with Starch4Kranz was limited and so we also compared our pipeline with that from LeafVeinCNN (Xu et al., 2021). Although LeafVeinCNN was designed for low‐magnification, unstained leaves from trees its automation was more comparable with our pipeline. When 27 images were subjected to the LeafVeinCNN pipeline (Xu et al., 2021) 3.5 min per image were required to run the CNNs but repeated manual input was required to obtain optimal thresholding. Moreover, compared with LeafVeinCNN (*r* = 0.74; Supporting Information: Figure S3B) the outputs from Starch4Kranz pipeline correlated more strongly with manual tracings (*r* = 0.95; Supporting Information: Figure S4A). LeafVeinCNN performed less well when vein densities were low (Supporting Information: Figure S3C), which may be because it was trained on data derived from leaves at lower magnification.

**Figure 4:**
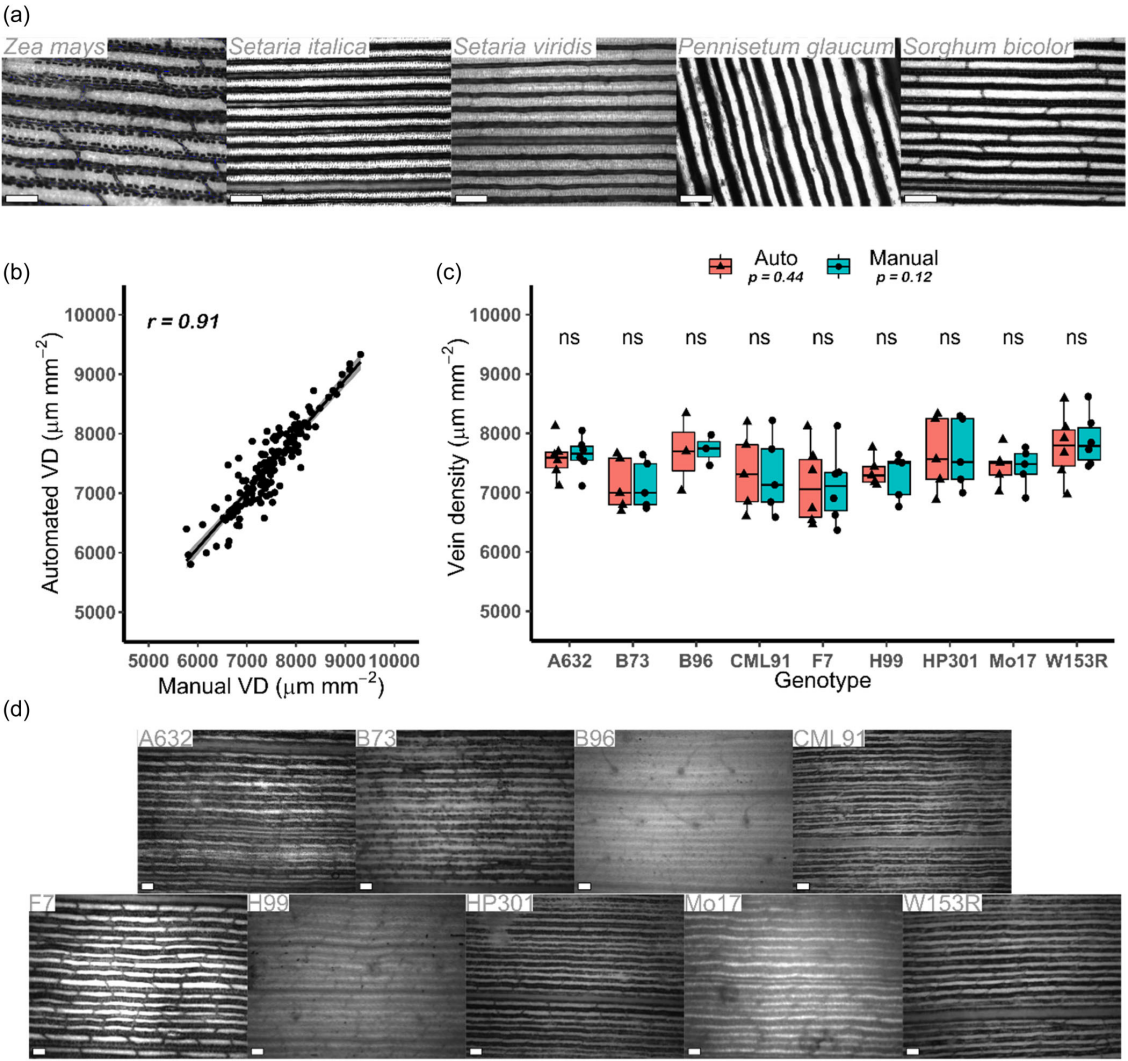
Starch staining of vein density in monocotyledons. (a) Preferential accumulation of starch revealed through staining of bundle sheath strands of the C_4_ monocotyledons *Zea mays*, *Setaria italica*, *Setaria viridis*, *Pennisetum glaucum* and *Sorghum bicolor*. (b and c) No significant differences between founder lines of maize were detected from either manual (red) or automatic (blue) methods. *p* Values determined by ANOVA and Student's *t* test. (d) Representative images of maize leaves stained for starch. Scale bars = 200 µm. *r*, Pearson's correlation coefficient; VD, vein density. [Color figure can be viewed at wileyonlinelibrary.com]

We found that subdividing an image into multiple sections, and capturing slightly out‐of‐focus images could enhance contrast between veins and background. This was of particular use for samples with high vein densities, or when staining was poor. As we found that some images did not allow accurate vein density to be determined, a manual check is incorporated into the pipeline (Figure 4b). Compared with other methods that require continual manual checking of images during processing (Bühler et al., 2015; Dhondt et al., 2012; Parsons‐Wingerter et al., 2014; Xu et al., 2021) Starch4Kranz saves time because it runs in the background across multiple images, and a manual check does not take place until the programme has finished. Machine learning techniques for image analysis are a useful tool for quantitative studies in plant sciences and have been employed to identify QTL associated with stomatal density in maize (Xie et al., 2021) and estimate heritability of vein density in sunflower (Earley et al., 2022). However, such approaches can require extensive computational understanding as well as power to build neural networks, and depending on the complexity of the phenotype may require manual annotation of thousands of images for network training (Moen et al., 2019; van Dijk et al., 2021). Thus, LeafVeinCNN is a powerful tool suitable for quantifying multiple traits associated with vein architecture, but Starch4Kranz offers a rapid and robust tool allowing vein density to be determined in C_4_ species. It also confirmed significant natural variation amongst F_2_ lines of *G. gynandra* (Figure 4c).

### 3.3 Natural variation in vein density was not detectable in founder lines of a maize MAGIC population

Aside from Grasviq (Robil et al., 2021) we noted a lack of automated methods for the assessment of vein density in monocotyledons. Because the preferential accumulation of starch in bundle sheath cells had first been reported in monocotyledons (Lunn & Furbank, 1997), we tested the NADP‐ME species *S. italica*, *S. viridis*, *P. glaucum* and *S. bicolor* as well as maize itself (Figure 4a) and then subjected various maize accessions to the Starch4Kranz algorithm. For this, an additional step was added to the pipeline to remove commissural veins (Supporting Information: Figure S5), which are not considered important components of Kranz anatomy (Langdale, 2011). We also noted that they stained for starch inconsistently likely because they are considered a simplified vascular tissue not encircled by bundle sheath cells (Sakaguchi & Fukuda, 2008; Sakaguchi et al., 2010). If commissural veins are of interest, and sufficient contrast obtained, then ‘auto’ can be selected as an input variable instead of ‘monocot’, or the ‘monocot_branch_length_to_keep’ can be set to 0 (see Section 2; Supporting Information: Table S1) and commissural veins will not be removed. Commissural veins were defined as the shortest branch stemming from a pixel with three branch points. Pattern recognition allowed these to be identified and removed (Supporting Information: Figure S5). The Python implementation of Starch4Kranz works slightly differently and detects commissural veins as the shortest Eulidean distance between two branch points and then removes this if it is below the ‘monocot_branch_length_to_keep’ variable (Supporting Information: Table S1). To ground‐truth this approach, manual tracings that did not include these veins were undertaken. Images from starch staining of nine founder lines of a MAGIC population from maize (Dell'Acqua et al., 2015) were subjected to Starch4Kranz. Estimates were highly correlated with manual analysis (*r* = 0.91; Figure 4a) but no statistically significant differences were observed between genotypes (*p* > 0.05; Figure 4b). When ran through the Python version the correlation was slightly weaker (*r* = 0.81; Supporting Information: Figure S2C) likely due to differences in commissural vein detection. Thus, Starch4Kranz can be used to quantify vein density in C_4_ monocotyledons in addition to dicotyledons (Figures 3 and 4; Supporting Information: Figure S6). In addition to this, we show that starch staining highlights the veins of both NAD‐ME and NADP‐ME species (Figures 1 and 4a). However, for the MAGIC maize founder lines studied here, we detected no significant differences in vein density (Figure 4c).

This finding emphasises the need to generate specific mapping populations designed for the trait of interest. *G. gynandra* F_2_ lines produced from a cross between two lines phenotypically distinct for vein density displayed abundant natural variation in this trait (Figure 3c).

Variation for vein density was not present in the MAGIC maize founders (Figure 4c) indicating that the MAGIC maize population (Dell'Acqua et al., 2015) is unlikely to be suitable for studying QTL underlying vein density. However, using a subset of lines from an association panel of 302 maize lines (Flint‐Garcia et al., 2005), Kolbe and Cousins (2018) did identify significant variation for vein density. Considering the ease and speed with which Starch4Kranz permits accurate quantification of vein density means that further screening of this association panel or other C_4_ mapping populations such as NAM populations of maize (McMullen et al., 2009; Yu et al., 2008) or MAGIC populations of sorghum (Ongom and Ejeta, 2017) could prove valuable for future detection of QTL controlling vein density.

In summary, we report a simple protocol allowing vein density to be determined from starch staining of vascular tissue in C_4_ species. Automation of this pipeline allowed rapid and accurate quantification of vein density. As increased vein density is considered an important trait allowing C_4_ photosynthesis (Sage, 2001), where intraspecific natural variation for this trait exists our approach could provide insight into mechanisms evolution has co‐opted to generate the complex C_4_ phenotype.

## Supporting information

Supporting information.

Supporting information.
