# Supplementary material for: A rapid method to quantify vein density in C4 plants using starch staining
Source: Plant Cell Environ. 2023 Jun 23;46(9):2928–38. doi: 10.1111/pce.14656 (PMC10947256; doi:10.1111/pce.14656)
Supplement: Supplementary file 1 — Supporting information. [file PCE-46-2928-s001.docx]

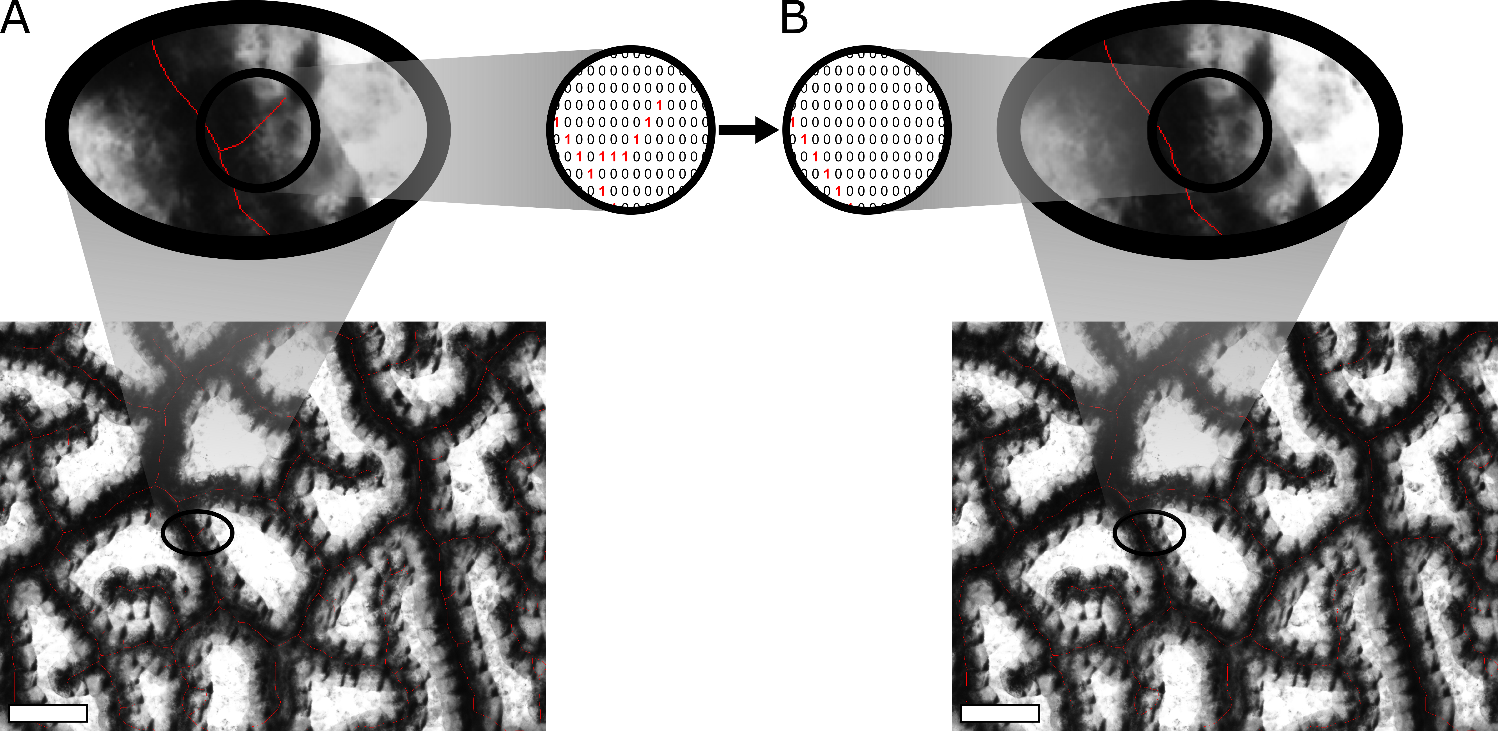


**Figure S1: Trimming of superfluous pixels. (A)** A branch end is recognized as a line of pixels with a single branch point and a single terminus. The binary code represents background (0) and vein (1). Note values of one are no longer present once after trimming **(B).** So, if the length of this line is below the trim_factor threshold, i.e., it is small and therefore not considered a vein but an array of superfluous pixels, it is trimmed. Scale bars represent 200$\mu m$.

**
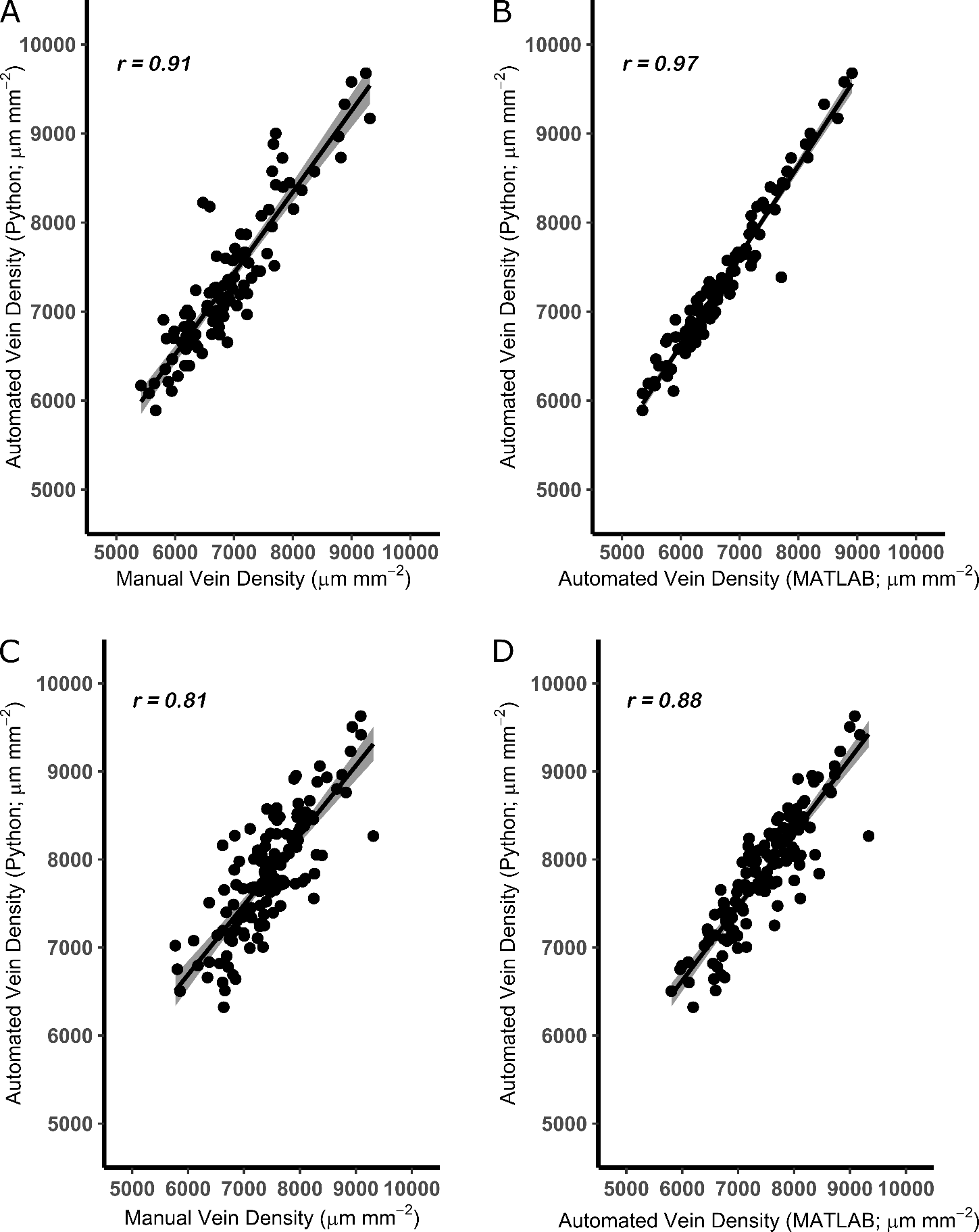
**

**Figure S2: Python version of Starch4Kranz is highly correlated with MATLAB version.** The correlation of vein density measured automatically for *G. gynandra* in Python versus **(A)** manual traces and **(B)** the MATLAB version. The correlation for vein density in maize when measured automatically in Python versus **(C)** manual traces and **(D)** the MATLAB version.


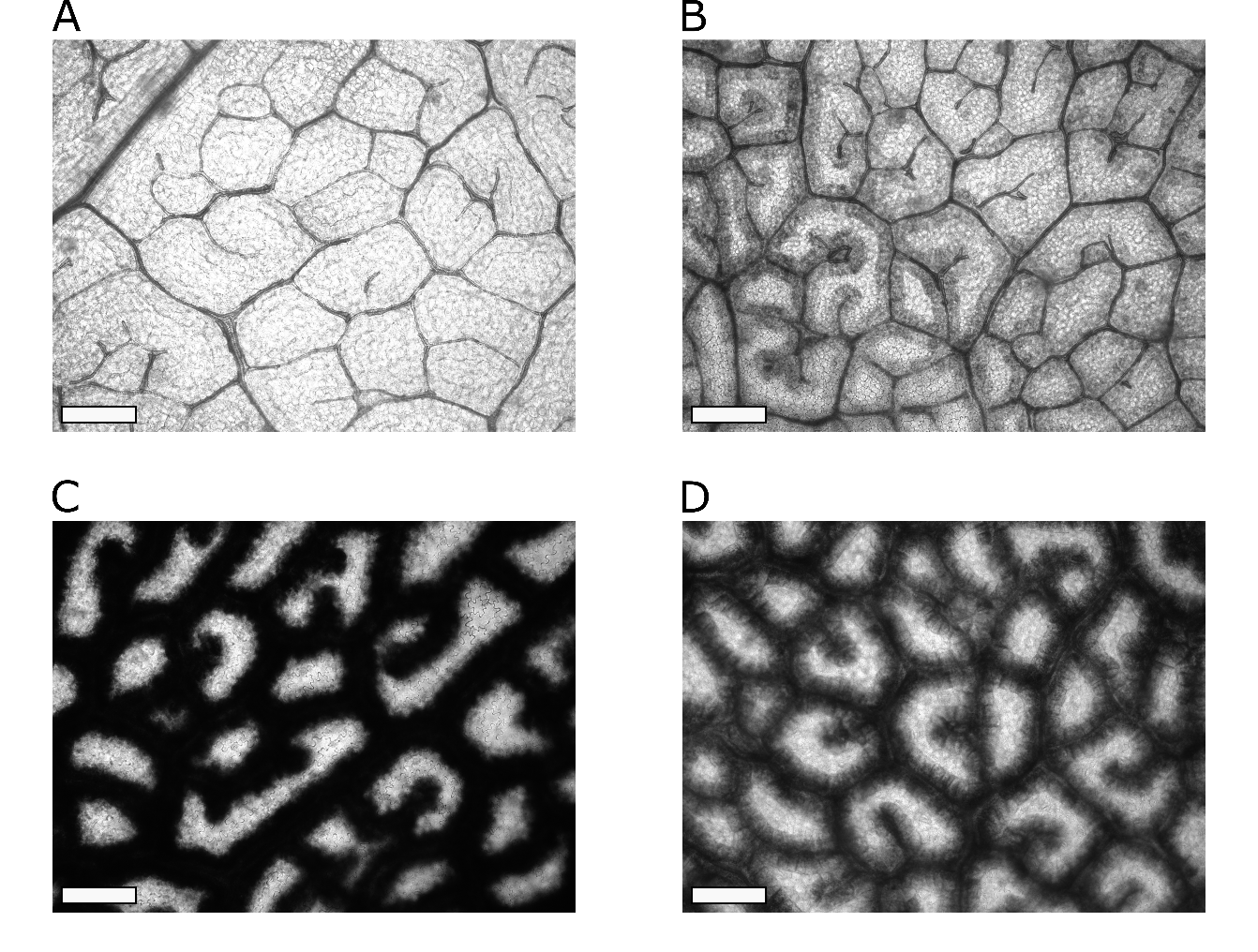


**Figure S3: Accumulation of starch in *G. gynandra* over a 16-hour photoperiod. (A)** At 0 hours into the photoperiod starch was not detectable. **(B)** By 6 hours into the photoperiod accumulation of starch was apparent. **(C)** By 9 hours into the photoperiod and at the end of the photoperiod **(D)** sufficient starch was detected to generate high-contrast images. Scale bars represent 200$\mu m$.

***
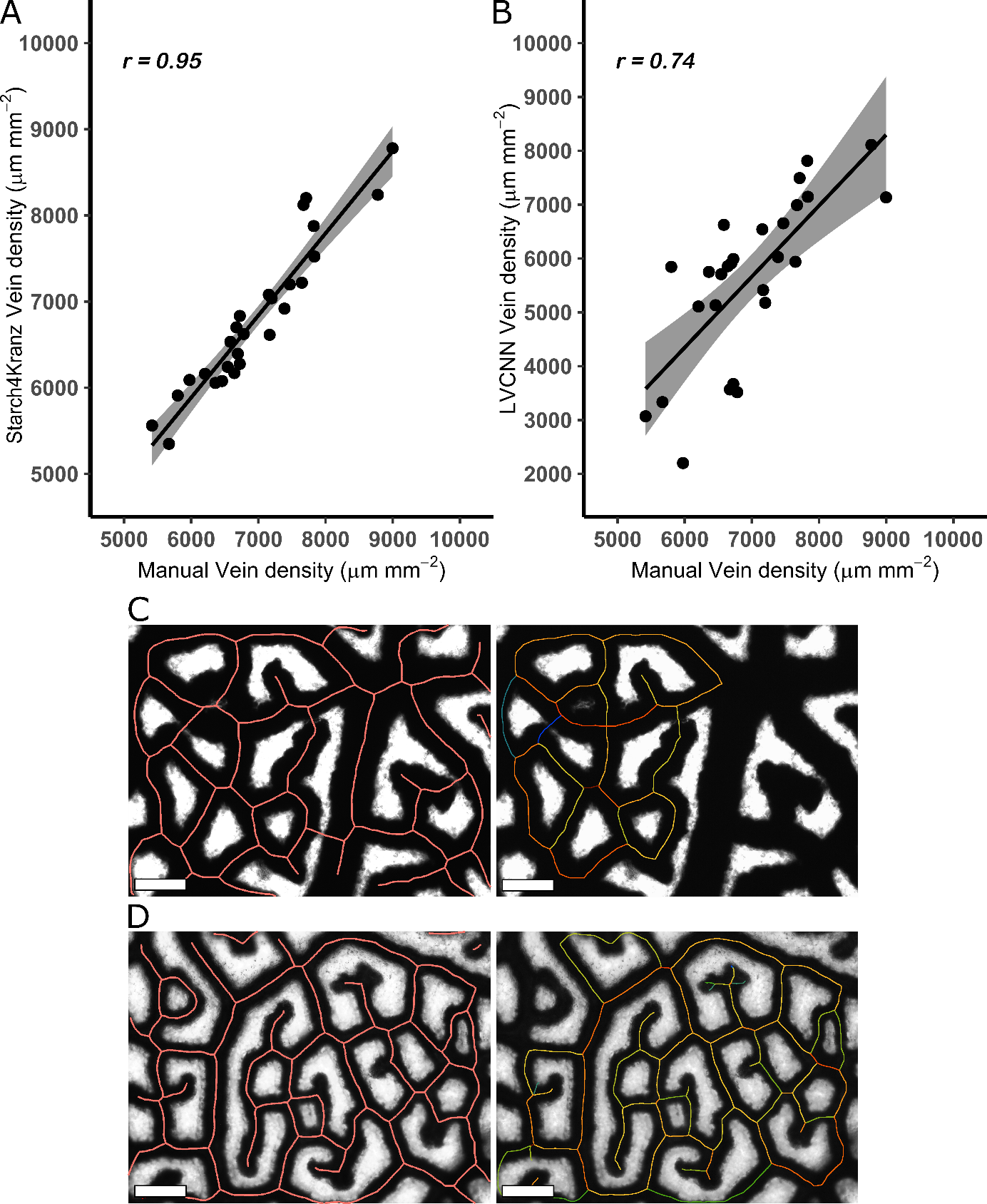
***

**Figure S4: Comparison between Starch4Kranz and LeafVeinCNN. (A)** Starch4Kranz correlated more strongly with manual traces compared to LeafVeinCNN **(B)**. Lower vein densities were detected less well by LeafVeinCNN **(B)** compared with Strach4Kranz **(D)**. $r$ = Pearson’s correlation coefficient; LVCNN – LeafVeinCNN. Scale bars represent 200$\mu m$. Skeletons have been thickened to improve their visibility.


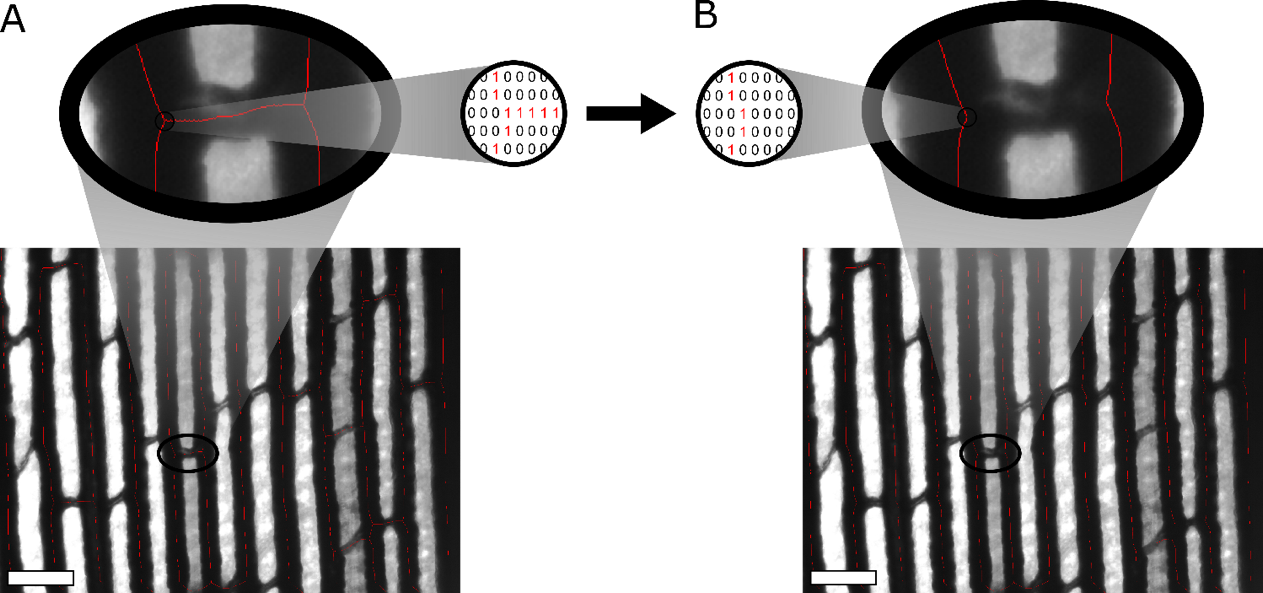


**Figure S5: Removal of commissural veins via pattern recognition. (A)** A commissural vein is recognized as being a pixel with three branch points. The binary code in the circle represents background (0) and vein (1). Note values of one are no longer present once commissural vein are removed **(B)**. Scale bars represent 200$\mu m$.

*
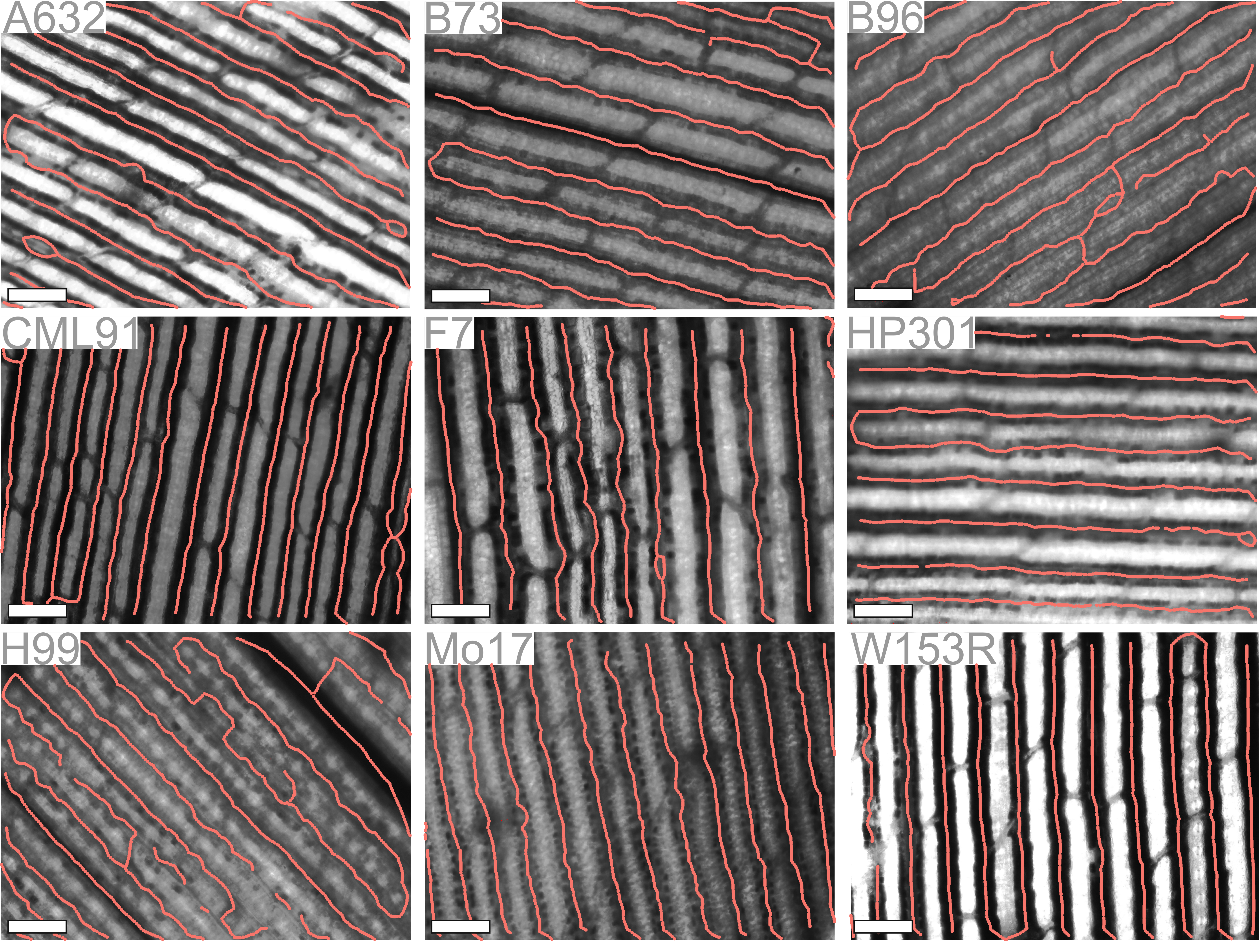
*

**Figure S6: Automated tracings for each accession of maize.** Representative outputs from Starch4Kranz on each of founder in the MAGIC maize population. Scale bars represent 200$\mu m$. Skeletons have been thickened to improve their visibility.
