# Supplementary material for: A rapid method to quantify vein density in C4 plants using starch staining
Source: Plant Cell Environ. 2023 Jun 23;46(9):2928–38. doi: 10.1111/pce.14656 (PMC10947256; doi:10.1111/pce.14656)
Supplement: Supplementary file 2 — Supporting information. [file PCE-46-2928-s002.docx]

**Table S1.** User inputs for Starch4Kranz.

| **Input** | **Use** |
| --- | --- |
| filename | File name of image to be processed; is looped through if using the “running_Starch4Kranz.m/py” script. |
| trim_factor | The length in pixels to trim superfluous ends. |
| pixel_length_um | The length of pixels in µm; is the scale for the image. |
| x_pixels | The number of pixels in the x dimension of the figure. |
| y_pixels | The number of pixels in the y dimension of the figure. |
| split_mode | Default is “auto”, but can be altered to “whole”, “split” or “monocot”, if you want to specify how the image is processed. |
| shave | When an image is split, it is cropped into 4 sub-images, to avoid the edge veins being measured twice, each image is cropped a certain number of pixels from the edge; this is defined by shave. Default is 40. |
| initial_blur_factor | The parameter given to the blur function. Default is 20. |
| branch_threshold | For when “split_mode = “auto””. If the number of branches following splitting is less than this value, then it likely would be best of to not be split (i.e. it has a lower vein density), hence will be processed as a whole image instead. Default is 125. |
| endpoint_threshold | For when “split_mode = “auto””. If the number of end points following splitting is less than this value, then it likely would be best of to not be split (i.e. it has a lower vein density), hence will be processed as a whole image instead. Default is 175. |
| sd_threshold | A higher standard deviation (sd) in the image implies higher contrast, as high contrasting images are best not split, if the sd of the grayscaled image is higher than this threshold it will be processed as a whole image. Default is 75. |
| trim_threshold | If following trimming based on the trim_factor, the ratio of number of branches present in the skeleton to total number of pixels present in the skeleton to is too high, then it should be trimmed based on the mode branch length instead. This input defines this cutoff. Default is 0.01. |
| mono_min_param | This is the length of pixels below which should not be removed as commissural veins. It is only considered if “split_mode = “monocot”” is selected. Default is 30. |
| monocot_branch_length_to_keep | This is the length of pixels above which should not be removed as commissural veins. It is only considered if “split_mode = “monocot”” is selected. If the user is interested in commissural veins, then they should set this parameter as 0. Default is 200. |
